# Genomic and taxonomic evaluation of 38 *Treponema* prophage sequences

**DOI:** 10.1186/s12864-024-10461-5

**Published:** 2024-06-01

**Authors:** Rachel Ridgway, Hanshuo Lu, Tim R. Blower, Nicholas James Evans, Stuart Ainsworth

**Affiliations:** 1 Department of Infection Biology and Microbiomes, University of Liverpool, Leahurst Campus, Chester High Road, Neston, Cheshire, CH64 7TE UK; 2 Department of Infection Biology and Microbiomes, University of Liverpool, Biosciences Building, Crown Street, Liverpool, L69 7BE UK; 3 Department of Biosciences, Durham University, Stockton Road, Durham, DH1 3LE UK; 4 Department of Infection Biology and Microbiomes, University of Liverpool, Liverpool Science Park IC2, 146 Brownlow Hill, Liverpool, L3 5RF UK

**Keywords:** Bacteriophages, Prophages, *Treponema*, Bioinformatics, *Treponema* phages, Genomic analysis, Comparative genomics

## Abstract

**Background:**

Despite Spirochetales being a ubiquitous and medically important order of bacteria infecting both humans and animals, there is extremely limited information regarding their bacteriophages. Of the genus *Treponema*, there is just a single reported characterised prophage.

**Results:**

We applied a bioinformatic approach on 24 previously published *Treponema* genomes to identify and characterise putative treponemal prophages. Thirteen of the genomes did not contain any detectable prophage regions. The remaining eleven contained 38 prophage sequences, with between one and eight putative prophages in each bacterial genome. The prophage regions ranged from 12.4 to 75.1 kb, with between 27 and 171 protein coding sequences. Phylogenetic analysis revealed that 24 of the prophages formed three distinct sequence clusters, identifying putative myoviral and siphoviral morphology. ViPTree analysis demonstrated that the identified sequences were novel when compared to known double stranded DNA bacteriophage genomes.

**Conclusions:**

In this study, we have started to address the knowledge gap on treponeme bacteriophages by characterising 38 prophage sequences in 24 treponeme genomes. Using bioinformatic approaches, we have been able to identify and compare the prophage-like elements with respect to other bacteriophages, their gene content, and their potential to be a functional and inducible bacteriophage, which in turn can help focus our attention on specific prophages to investigate further.

**Supplementary Information:**

The online version contains supplementary material available at 10.1186/s12864-024-10461-5.

## Background

Bacteriophages (phages) are viruses that are obligatory intracellular parasites of bacteria [1]. These important bacterial predators are the most abundant biological entities on Earth with the global population of phages estimated to be around 10^31^ [2, 3]. Despite this well-acknowledged abundance, as of August 2023, a comparatively small number, approximately 44,000 phage genomes, have been officially documented with NCBI [4], with the majority of all deposited phage sequences from representatives of the *Caudoviricetes* class of tailed phages [5]. Phages exhibit different lifestyles, they can be lytic, swiftly killing their bacterial host cells upon replication and release, or lysogenic, integrating their genome into the host DNA, forming a prophage. Additionally, phages may adopt pseudolysogeny, often in conditions that cause suboptimal growth of the host bacteria, triggering a stage of stalled development during which neither phage genome replication nor prophage formation occurs [6, 7]. Chronic infection lifestyles also exist for filamentous phages, which slowly release from the host cell over an extended period without causing cell death [8].

In the lysogenic state, integrated genomes are transmitted to daughter cells through bacterial replication. Prophages can manifest in functional or nonfunctional form [9], in most cases the lysogenic cycle also allows for the exit into the lytic cycle upon induction, so called inducible phages, able to form infectious particles. Prophages may also be nonfunctional or cryptic phages, which harbour deletions, insertions and rearrangements that render them unable to complete the lytic cycle [10].

Prophages have been demonstrated to have substantial influence on their host genomes and are recognised to be key drivers of evolutionary changes in prokaryotic communities, often by enabling genome plasticity and altering host phenotypes [11]. In particular, prophages can be associated with increased virulence of pathogens, through the ability to encode toxins, antibacterial resistance and alter host bacterial properties relevant to all stages of the infectious process [12].

Due to increasing bacterial resistance to antibiotics and a dearth of new antibiotics coming onto the market, there is increasing interest and research in phage therapy to combat this major threat to public health [13]. Compared to temperate phages, lytic phages have been traditionally sought after as therapeutic agents, as they are lethal to bacteria akin to antibiotics and likely easier for approval as a treatment for bacterial infections [14]. However, temperate phages have also been investigated for phage therapy purposes; following genetic manipulation to remove the genes essential for lysogeny [15–18], and after the discovery of spontaneous mutations, preventing lysogeny among environmental isolates [19, 20]. These former temperate phages have been used to successfully treat bacterial infections in vivo [15]. There are also other potential options to explore, for example, using temperate phages to introduce, by lysogeny, genes conferring sensitivity to antibiotics that previously the pathogen had been resistant to [21]. Another study [22] demonstrated that *Clostridium difficile* phages despite containing integrases, all accessed the lytic pathway and so have potential as a future treatment even though they have the ability to access the lysogenic cycle. Currently, these non-lytic examples are not preferred by regulatory bodies for application of phage therapy, however, all areas warrant investigation.

Our understanding of phage infections in spirochetes is notably limited when compared to other prokaryotes. In particular, our knowledge of phages infecting *Treponema* species is still in its infancy, with only a scant number of reports, mostly observations in electron microscopy images, documenting such occurrences [23–28]. To our knowledge, only one *Treponema* prophage has been successfully induced and characterised in any detail, phage td1, from the genome of *Treponema denticola* [28].

The genus *Treponema* is of significant medical importance for both humans and animals, encompassing pathogens responsible for human and veterinary diseases such as syphilis, yaws, bejel, periodontal disease, *Leporidae* syphilis, and bovine digital dermatitis disease [29, 30], as well as being associated with various necrotising infections, such as Noma [31]. Historically, the comprehensive study of treponemes and their associated biology has faced challenges due to their fastidious nature, which makes isolation and cultivation difficult [32]. However, in recent times, cultivation of treponemes has become more common place due to the ability to provide their specific conditions [33], which has made the study of treponemes and their phages more feasible.

The post genomic era offers an opportunity to characterise spirochete-infecting phages that are present as prophages in available bacterial genomes in detail. There are a substantial number of treponeme species, isolated from diverse environments, whose complete genomes have been sequenced and can be analysed for the presence of phages [34].

The objective of this study was to use a bioinformatic approach to examine 24 complete *Treponema* genomes available when NCBI was queried (11th December 2022), to identify and characterise treponeme prophages at the genomic level.

## Results

### Identifying putative prophages in genomes of *Treponema*

**Fig. 1:**
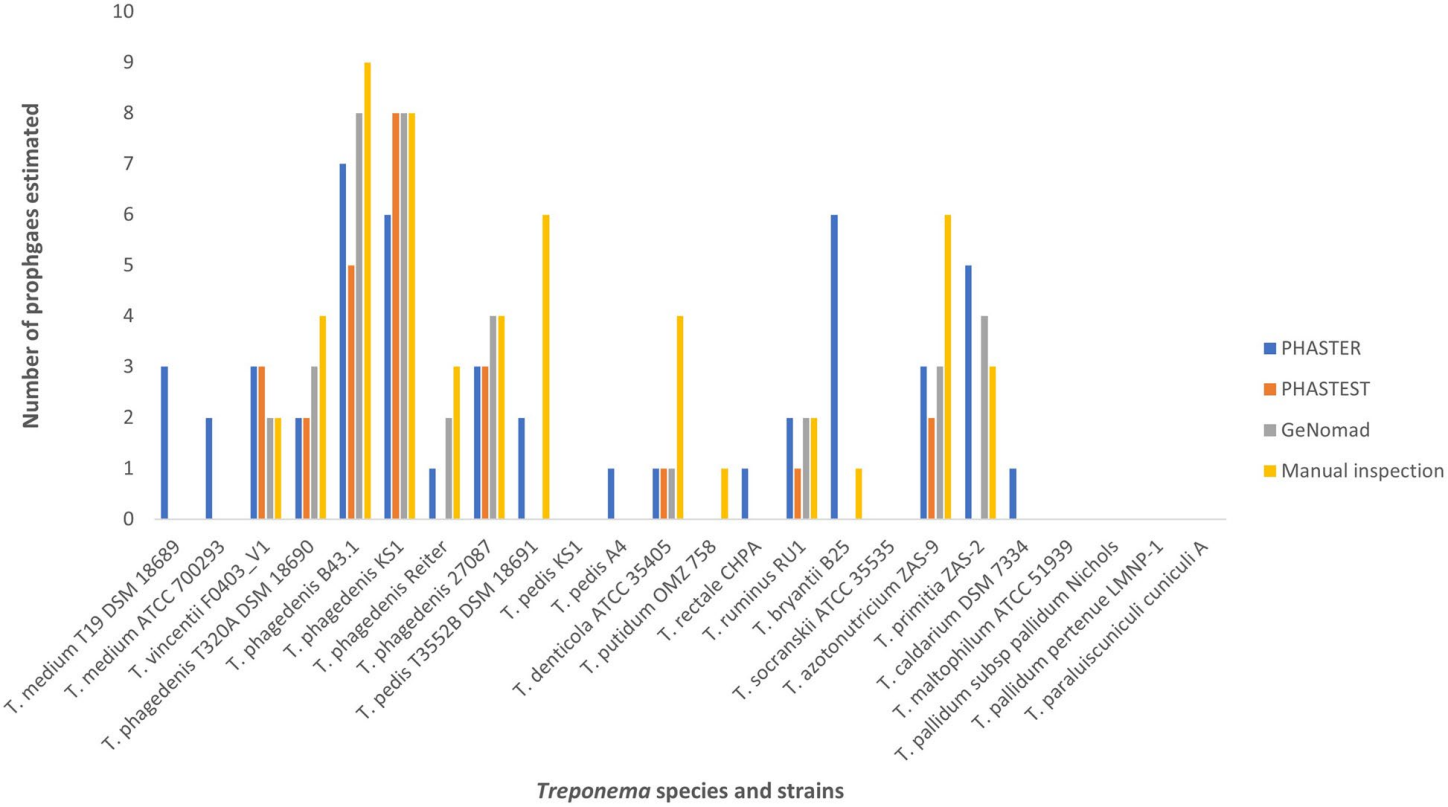
Bar chart to show the number of prophage regions estimated by each detection method; PHASTER, PHASTEST, geNomad and manual inspection

The dataset investigated composed of 24 completed *Treponema* genomes representing 16 Treponemal species, accessed via GenBank. A combination of tools is required when detecting novel phage [35], therefore, PHASTER, PHASTEST and geNomad were used to identify prophage-like elements within these genomes, as well as a comprehensive manual review of each treponemal genome as per the criteria stated in the methods. PHASTER identified 49 regions, PHASTEST identified 25 regions and geNomad identified 37 prophage regions, while manual inspection identified 52 regions (Fig. 1). All the identified regions were then interrogated by CheckV, with any sequences failing CheckV verification as a putative prophage sequence removed. This pipeline resulted in 38 prophage sequences that had been identified by at least two prophage detection approaches, except for the prophage detected in *T. bryantii*, which was identified by manual inspection only. PHASTEST was able to identify putative *att* sites for seven prophages. The sequence provided for the *att* sites for the prophage in *T. denticola* differs from the predicted td1 phage *attB* site by Mitchell et al. [28] after they were able to induce the prophage.

Approximately half (13/24, 54%) of the treponemal genomes interrogated for the presence of prophage, did not contain any potential prophage regions, while the remaining genomes (11/24, 46%) yielded 38 putative prophage regions. The number of prophage-like sequences varied from one to eight per genome, with lengths ranging from 12.4 kb to 75.1 kb and encoding between 27 and 171 potential protein coding sequences. To provide context, the smallest known tailed phages measure approximately 11.5 kb for podoviral morphology [36], 21 kb for siphoviral morphology [37], and 30 kb for myoviral morphology [38]. The prophage regions exhibited an average guanine plus cytosine (GC) content of 41.6%, closely resembling the average GC content of their respective *Treponema* host strains (Table 1). *Treponema phagedenis* B43.1 contained the most prophage DNA in its genome at 12.8% (eight prophage regions).

**Table 1:** Three distinct clusters of treponeme prophages and description of each prophage

^**Cluster**^	^***Treponema*strain**^	^**Prophage name**^	^**CheckV quality**^	^**Length (kb)**^	^**CDS**^	^**GC%**^	^Predicted morphology*^	^**Defence Systems/morons**^	
^**A**^	^*T. phagedenis*B43.1^	^B43P7^	^medium−73%^	^64.2^	^84^	^40.9^	^Myoviral^	^None found^	
	^*T. phagedenis*Reiter^	^ReiterP1^	^medium−63%^	^52.5^	^75^	^40^	^Myoviral^	^None found^	
	^*T. phagedenis*^ ^ATCC 27087^	^27087P2^	^medium−84%^	^67.6^	^88^	^40^	^Myoviral^	^None found^	
	^*T. phagedenis*KS1^	^KS1P5^	^medium−81%^	^65.9^	^93^	^39.5^	^Myoviral^	^None found^	
	^*T. phagedenis*T320A^	^T320AP2^	^low−41%^	^54^	^71^	^38.9^	^Myoviral^	^None found^	
	^*T. phagedenis*ATCC 27087^	^27087P3^	^high−91%^	^73.2^	^98^	^39.5^	^Myoviral^	^None found^	
	^*T. phagedenis*B43.1^	^B43P4^	^high−91%^	^73.5^	^81^	^39.8^	^Myoviral^	^None found^	
	^*T. phagedenis*B43.1^	^B43P5^	^high−91%^	^73^	^81^	^39.4^	^Myoviral^	^None found^	
	^*T. phagedenis*KS1^	^KS1P4^	^medium−60%^	^65.9^	^93^	^39.5^	^Myoviral^	^None found^	
	^T. phagedenis KS1^	^KS1P7^	^medium−74%^	^62.1^	^95^	^40.8^	^Myoviral^	^None found^	
^**B**^	^*T. phagedenis*B43.1^	^B43P1^	^low−42%^	^40.6^	^61^	^40.1^	^Siphoviral^	^None found^	
	^*T. phagedenis*B43.1^	^B43P2^	^low−44%^	^46.8^	^74^	^39.8^	^Siphoviral^	^Thoeris_type1^	^ThsB1^ ^ThsA1^
	^*T. phagedenis*KS1^	^KS1P8^	^low−45%^	^39.5^	^75^	^39.2^	^Siphoviral^	^None found^	
	^*T. phagedenis*T320A^	^T320AP3^	^low−48%^	^53.1^	^97^	^38.8^	^Siphoviral^	^None found^	
	^*T. phagedenis*KS1^	^KS1P1^	^low−40%^	^33.4^	^57^	^39.9^	^Siphoviral^	^None found^	
	^*T. phagedenis*KS1^	^KS1P2^	^low−36%^	^52.9^	^93^	^39.7^	^Siphoviral^	^None found^	
	^*T. phagedenis*KS1^	^KS1P3^	^low−40%^	^33.9^	^56^	^39.5^	^Siphoviral^	^None found^	
	^*T. phagedenis*B43.1^	^B43P3^	^low−43%^	^36.1^	^56^	^39.1^	^Siphoviral^	^None found^	
	^*T. phagedenis*B43.1^	^B43P8^	^medium−64%^	^53.6^	^87^	^37.7^	^Siphoviral^	^RM_type_II^	^MTase_II^ ^REase_II^ ^MTase_II^
	^*T. phagedenis*ATCC 27087^	^27087P1^	^medium−42%^	^33.5^	^51^	^39.6^	^Siphoviral^	^None found^	
^**C**^	^*T. phagedenis*B43.1^	^B43P6^	^medium−61%^	^47.6^	^77^	^39.2^	^Siphoviral^	^None found^	
	^*T. phagedenis*T320A^	^T320AP1^	^medium−80%^	^65.3^	^88^	^39.6^	^Siphoviral^	^RM_type_II^	^REase_II^ ^MTase_II^
	^*T. phagedenis*ATCC 27087^	^27087P4^	^medium−67%^	^52.9^	^86^	^38.8^	^Siphoviral^	^None found^	
	^*T. phagedenis*KS1^	^KS1P6^	^medium−57%^	^44.2^	^72^	^39.8^	^Siphoviral^	^None found^	

The table shows the prophages in each cluster, the size, the GC content, whether any defence systems were located and what morphology is alluded to from the features of the prophages

^*^The predicted morphology of the prophage like sequences is based on the presence of a tail sheath protein (indicating myoviral morphology) and a tail length tape measure protein (indicating siphoviral morphology)

### Genome-based phylogeny of the Treponeme infecting prophages

**Fig. 2:**
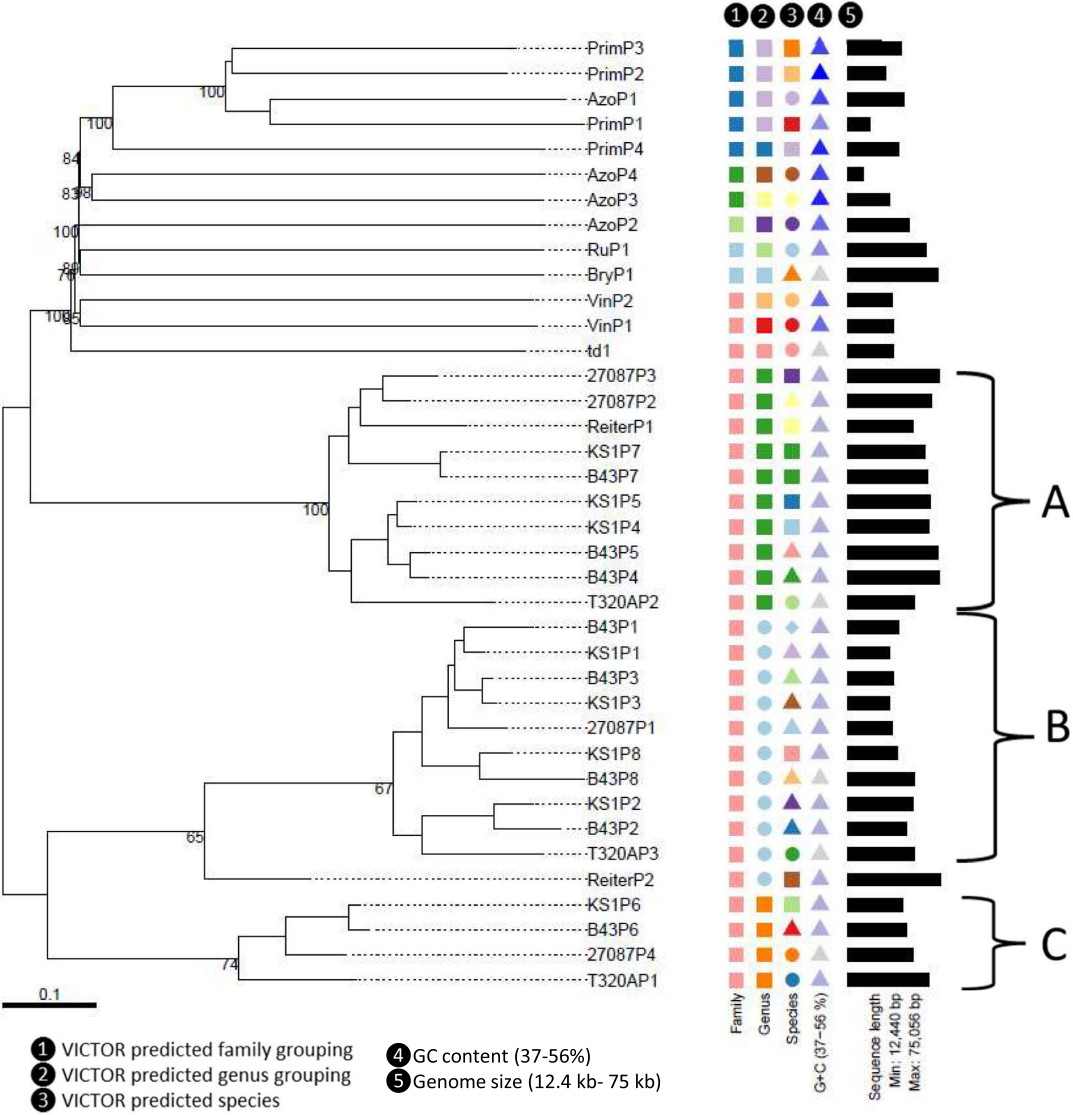
Phylogenetic tree generated by VICTOR using the predicted genome sequences of the 38 different prophage regions. Three clusters of prophages were identified with genetic similarities (**A**, **B** and **C**). The colours of the key indicate which prophages are predicted by VICTOR to be of the same family, genus, or species, as well as the GC content and genome size. Treponemal species are designated at the start of the phage name: T. azotonutricium – Azo, T. primitia – Prim, T. ruminus – Ru, T. bryantii – Bry, T. denticola – td, T. vincentii- Vin, T. phagedenis – KS1, B43, 27,087, Reiter, T320A

Multiple bioinformatic methods were then used to characterise and investigate the genomic diversity of the prophages. A phylogenetic tree of the 38 prophage regions was created by VICTOR (Fig. 2) using intergenomic distances based on protein–protein BLAST comparisons of the whole viral proteomes to infer evolutionary relationships between the predicted prophages. The genome comparison of all the prophage regions highlighted three clusters of the same genus composed of at least four prophage sequences, all of which derived from *T. phagedenis* strains isolated from either bovine digital dermatitis lesions or human samples and from different geographical regions. Cluster A comprises ten prophage regions, another ten prophage sequences are included in cluster B and cluster C incorporates four prophage regions with genetic similarities. A fourth less closely related cluster, but of the same genus can be seen at the top of the figure, consisting of three prophages from *T. primitia* and one prophage from *T. azotonutricium*. With the exception of ReiterP2, which appears to be from a lineage related to cluster B, the remaining identified prophage sequences appear to show very little to no genetic relationships to any of the *Treponema* prophage sequences identified.

**Fig. 3:**
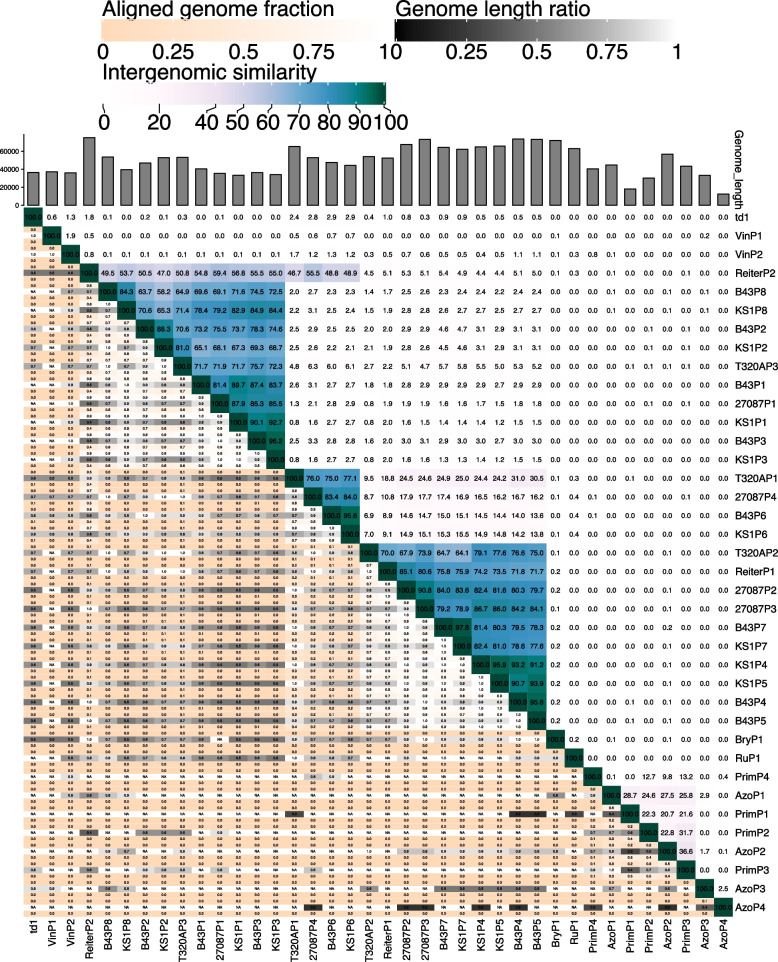
Intergenomic similarity analysis of the 38 Treponema prophage sequences using VIRIDIC generated a heatmap incorporating intergenomic similarity values (right half) and alignment indicators (left half and top annotation). In the right half, the more closely-related the genomes, the darker the colour and the numbers represent the similarity values for each genome pair, rounded to the first decimal. In the left half, the darker colours emphasize low values, indicating genome pairs where only a small fraction of the genome was aligned (orange to white colour gradient), or where there is a high difference in the length of the two genomes (black to white color gradient). The reward and penalty scores for matching and mismatching bases, respectively, were set to 1 and − 2, the same as the default parameters of the NCBI_BLASTN. The species and genus threshold values were set to 95% and 70% intergenomic similarities, respectively

The 38 prophage sequences were then analysed via VIRIDIC (Fig. 3), to provide intergenomic similarity values, which is the standard used by the International Committee on Taxonomy of Viruses (ICTV) to classify phage at the genus or species level [39]. Notably, the results identified the same three *T. phagedenis* clusters identified by VICTOR (Fig. 2), highlighted on the right-hand side heat map in Fig. 3 in blue and green. VIRIDIC has the benefit of showing the percentage similarity of the genome alignment, with some of the genomes in these clusters being as closely related as 96% similarity (range 58.2%—96.2% similarity) (Fig. 3). VIRIDIC established the less significant cluster identified in VICTOR consisting of PrimP1, PrimP2, PrimP3 and AzoP1 (Fig. 2) as having between 21.6% and 31.7% similarity and that ReiterP2 had between 46 to 59% similarity to the prophage regions in cluster B.

### Proteome-based classification of the treponeme infecting prophages

**Fig. 4:**
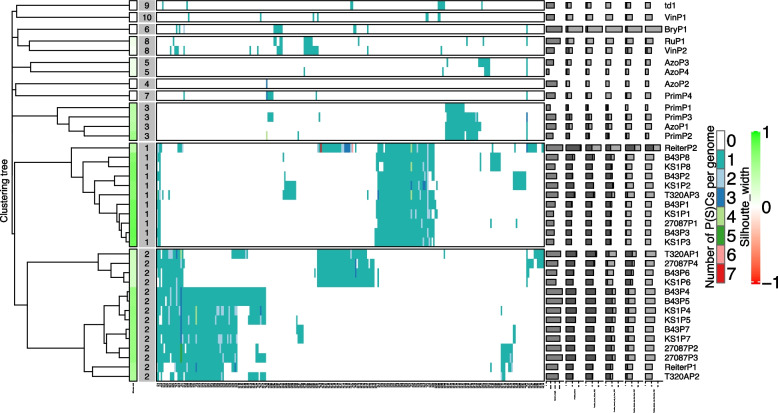
Integrated visualization of viral clustering by Virclust. The visual components are a hierarchical tree based on intergenomic distance to the far left, followed by silhouette width colour-coded in a range from -1 (red) to 1 (green) to show the separation distance between the resulting clusters, viral genome cluster (VGC) ID – 1 = cluster C, 2 = clusters A and C, 3 = cluster D), a heat map representation of the protein clustering in the prophage genomes and viral genome specific statistics: genome length, proportion of proteins shared (dark grey) with any other genomes in the dataset, proportion of protein (super) clusters (P(S)Cs) shared in its own VGC, proportion of PCs showed only in its own VGC, proportion of PCs shared also outside its own VGC, and the proportion of PC shared only outside own VGC

Virclust analysis provides visualisation and details of protein clustering in the different prophage sequences, as well as inferring phylogenetic relationships (Fig. 4). These results similarly identified the same three main clusters as VICTOR (Fig. 2) and VIRIDIC (Fig. 3) and can easily be seen on the heat map representation of protein clustering (cluster B = 1, clusters A and C = 2).

**Fig. 5:**
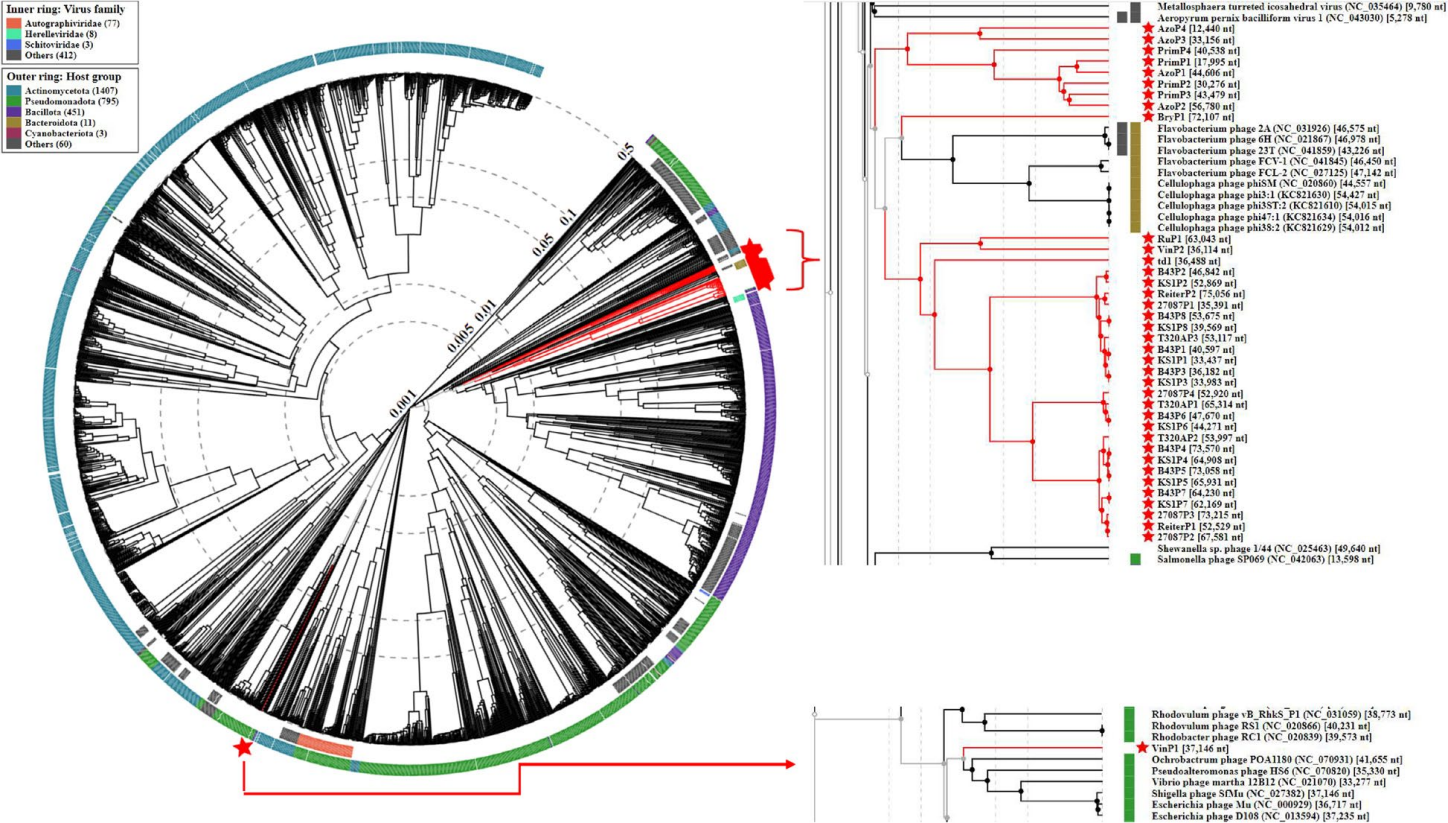
ViPTree circular proteomic tree of related dsDNA viruses with prokaryotic hosts. Submitted prophage-like sequences are highlighted in red

All 38 prophage sequences were submitted to ViPTree, which uses the same protein BLAST comparison method as VICTOR to determine the phylogenetic positioning against a global dsDNA viral reference database. This analysis resulted in 2837 entries in the final tree and identified all the putative *Treponema* prophages to be very closely clustered with one exception, VinP1 (Fig. 5). This prophage stands out among the 37 others, apparently being more closely related to *Vibrio* and *Escherichia* phages than all the other treponemal prophages identified in this study.

Clusters A, B and C share common lineages, featuring RuP1, VinP2, td1, and notably, the inclusion of ReiterP2 into cluster C, highlighting its close association with 27087P1. ViPTree also grouped eight prophages which had not been identified as belonging to a cluster as a further distinct lineage. The remaining unassigned prophage, BryP1 belonged to a lineage which appears to be more closely related to *Flavobacterium* and *Cellulophaga* phages (Fig. 5).

### Characterisation of the three main *Treponema* prophage clusters

**Fig. 6:**
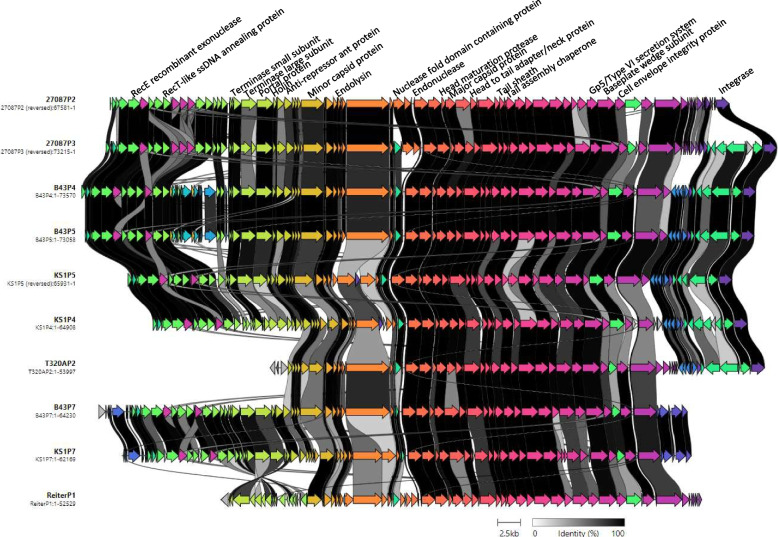
Comparative genome alignment of prophages comprising cluster A. Phage genomes are presented alongside their designated name and genome length. Coding sequences are represented by arrows coloured to reflect homologous groups identified by Clinker and are linked by grey bars shaded to represent the percentage amino acid identity, as indicated in the legend

**Fig. 7:**
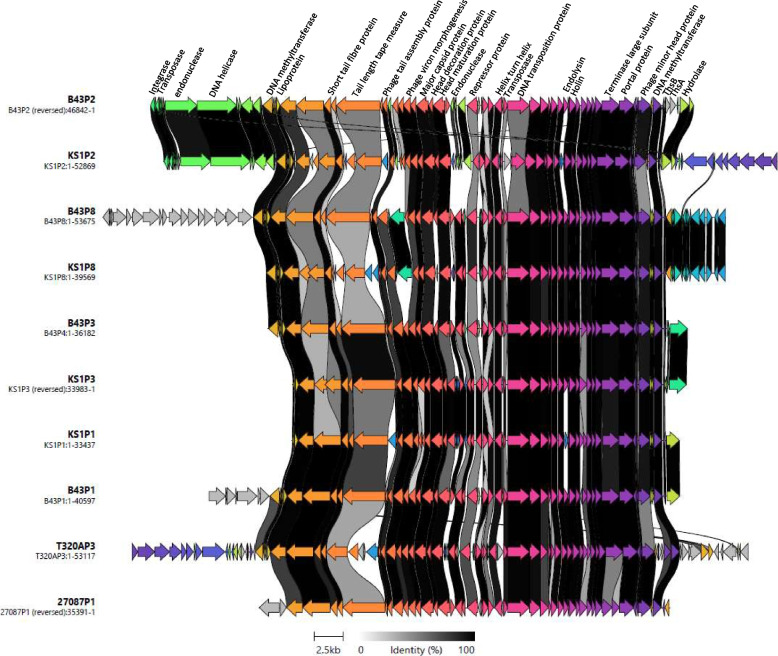
Comparative genome alignment of prophages comprising cluster B. Phage genomes are presented alongside their designated name and genome length. Coding sequences are represented by arrows, coloured to reflect homologous groups identified by Clinker, and are linked by grey bars shaded to represent the percentage amino acid identity, as indicated in the legend

**Fig. 8:**
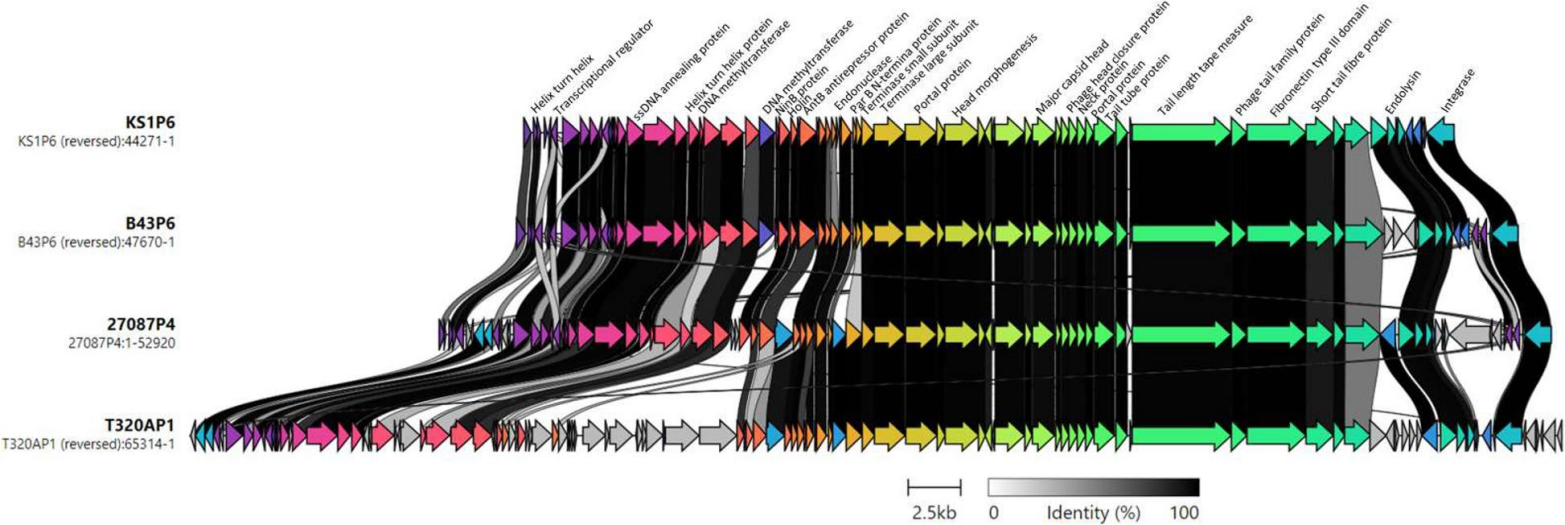
Comparative genome alignment of prophage comprising cluster C. Phage genomes are presented alongside their designated name and genome length. Coding sequences are represented by arrows, coloured to reflect homologous groups identified by Clinker, and are linked by grey bars shaded to represent the percentage amino acid identity, as indicated in the legend

The 24 prophage sequences which formed the three clear primary *Treponema* prophage clusters from *T. phagedenis* were further selected for in depth analysis (Table 1). A visual alignment of the prophages in each cluster was created using Clinker (Figs. 6, 7 and 8). PADLOC was used to identify any anti-viral defence mechanisms and PhageLeads and Pharokka were used to identify any virulence genes or antimicrobial resistance genes within the prophages which could be of benefit to the host bacteria.

#### Cluster A

There were ten putative prophages identified in cluster A, ranging from 52.5-73kb in length and encoding 71 to 102 protein coding sequences (Fig. 6). All prophages include a tail sheath encoding protein and so are likely to be of myoviral morphology. [40, 41]. Six prophages (27087P2, 27087P3, KS1P4, KS1P5, B43P4 and B43P5) include an integrase, a terminase and several structural conserved protein domains in the correct order (terminase – portal – protease – scaffold – major head shell (coat) protein – head/tail joining proteins – tail shaft protein – tape measure protein – tail tip/baseplate proteins – tail fibre) and so have the potential be intact [9, 42]. However, CheckV results indicated only 27087P3, B43P4 and B43P5 as high quality, at 91% complete and 73kb in length, while prophages 27087P2, KS1P4 and KS1P5 are shorter (66-67kb) and were considered medium quality. Prophage T320AP2 contains an integrase but no terminase and was considered low quality by CheckV and prophages ReiterP1, B43P7 and KS1P7 contain a terminase but no integrase and were considered medium quality by CheckV (Fig. 6). PADLOC identified only Methyltransferase proteins in B43P4, B43P5, KS1P4, KS1P5, 27087P3 and T320AP2 and no virulence or antibiotic resistance genes were detected by Pharokka or PhageLeads.

#### Cluster B

Cluster B includes ten prophage regions, ranging from 33.4–53.6 kb, encoding 43–93 protein coding sequences (Fig. 7). All ten genomes possessed a tail tape measure encoding protein sequence of various lengths, ranging from 0.9 to 3.75 kb, indicating likely siphoviral morphology [40, 41]. The first 17 protein coding sequences of B43P8 (CDS FUT79_RS13705 to FUT79_RS13630 in *T. phagedenis* B43.1) appear not to be present in any of the other prophages in the cluster. CDS 1 (FUT79_RS13705) is a helix-turn-helix protein, CDS 4 (FUT79_RS13690) in an integrase, CDS 9, 14 and 18 (FUT79_RS13665, FUT79_RS13650, FUT79_RS13630) are all DNA methyltransferase proteins.

CheckV results identified all sequences in this cluster as low to medium quality (Table 1). Despite examining the wider bacterial genome on either side of these sequences, no further phage coding sequences were identified. PADLOC identified a Thoeris type I system in B43P2 (CDS 4 and 5, Fig. 7) and a restriction modification (R-M) type II system in B43P8 (CDS 10 and 12). Using Uniprot, the R-M system in B43P8 was found to have the largest percentage identity to a restriction endonuclease (REase) (85% identity) and methyltransferase (MTase) (88.9% identity) in *Selenomonas sputigena*, an anaerobic Gram-negative bacteria.

#### Cluster C

Four prophages were identified in cluster C (Fig. 8) with a range of 44.2—65.3kb in length and encoding 72 to 88 protein coding sequences with all sequences being considered medium quality by CheckV (Table 1). The four prophage sequences have the same length tail length tape measure protein of 4718 bases, indicating potential siphoviral morphology. T320AP1 has a short section of genome dissimilar to any other prophage in the cluster (CDS 70–84 (CDS C5078_00805 – C5078_00770 in *T. phagedenis* T320A bacterial genome) (Fig. 8). Only CDS 73 was identified as a likely phage protein (phage family protein) by UniProt. PADLOC identified an R-M type II system in T320AP1 (CDS 70 and CDS 72). UniProt identified the MTase to be more similar to *Alysiella crassa* and *Prevotella corporis* modification methylase EcoRI, sharing 67.8% and 60.4% identity respectively. Both are Gram negative bacteria, *Alysiella* being motile and aerobic and *Prevotella*, anaerobic and non-motile. The REase was found to be most alike to a *Campylobacter hominis* nuclease at 71.7% identity, another Gram negative, motile bacterial species.

## Discussion

Despite the ubiquitous nature and medical significance of the genus *Treponema* [43], surprisingly little is known about its phages. In the current study we sought to develop a foundation knowledge of a subset of phages infecting *Treponema* through bioinformatic characterisation of prophages present in the genomes of 24 *Treponema* isolates of varying species from diverse environments.

Four prophage identification methods were used in this study, as well as the use of CheckV, to improve the accuracy of prophage prediction. This was further supplemented by four different programs for virus-based classification, each with differing strengths, which also provided further supportive evidence for confidence in the identification through recognising similar predicted phage clusters. Through this workflow, examination of *Treponema* genomes yielded 37 previously uncharacterised prophage regions (38 in total), with three clusters (named A, B and C) of closely related phages.

It is notable that the closely related phages from clusters A, B and C are all present in the same species, *T. phagedenis*. Three of the *T. phagedenis* strains examined in this study were isolated from bovine digital dermatitis lesions and are considered pathogenic, while the remaining two strains are human and considered saprophytic and nonpathogenic [34]. Examined *T. phagedenis* genomes to date appear to have less antitoxin systems compared with other *Treponema* species [34], which may make *T. phagedenis* more susceptible to larger prophage burdens.

Based on the presence of specific tail-structure encoding genes, all the putative prophages identified are predicted to have a myoviral or siphoviral morphology. In 2022, the ICTV introduced significant updates to the phage classification system [5]. As a consequence of these revisions, *Treponema* phage td1 [28], the sole treponema phage documented to have the excised prophage DNA detected to date and the rest of the putative prophages identified in this study belong to a yet undefined order.

In addition to previously demonstrated induction of prophages from *T. phagedenis* Reiter [26] and *T. denticola* [28], the observed genomic characteristics of the identified prophages suggest that several may have retained the functional capacity to form infectious particles.

However, it is noteworthy that examination of prophages within each cluster display considerable differences in size, indicating some may now be cryptic through deletion of prophage coding regions. Although, co-evolution with its host bacterium may mean that bacterial genes integrate into the prophage genome or that redundant genes are lost from the prophage during replication, resulting in changes in genome size of prophages from different bacterial strains [44].

It is notable that some genomes in this study encoded a substantial number of prophages. *T. phagedenis* B43.1 and *T. phagedenis* KS1 harboured the most prophage DNA with greater than 10% of their genome being of prophage origin. Other species have been noted to possess prophages constituting up to 20% of their total genome [9]. Fitness benefits can be provided to hosts for harbouring prophages, including superinfection exclusion, provision of antibiotic resistance and various virulence factors [45]. Whilst neither virulence nor antibiotic resistance genes were detected in any of the treponema prophages in this study via Pharokka and PhageLeads, PADLOC did detect three prophage regions containing anti-phage defence systems, providing the host with protection against further phage infection, favouring both the host and the prophage [46]. Prophages T320AP1 from cluster B and B43P9 from cluster C included a R-M type II defence system and B43P2 from cluster C contained a Thoeris defence system. The Thoeris system is an example of an abortive infection system comprising of two proteins; ThsB has a toll/interleukin-1 receptor (TIR) domain, which is activated by phage infection and produces signaling molecules. This activates ThsA, which contains a domain that binds to nicotinamide adenine dinucleotide (NAD^+^), causing hydrolysis, leading to depletion of the NAD^+^ pool and cell death [47, 48].

Conversely to the high prophage burdens of some strains analysed in this study, thirteen treponeme genomes were apparently completely void of any prophage-related sequences. This includes the three *T. pallidum* genomes, which were expected to be devoid of extraneous DNA due to their extremely limited genomes and dependency on their hosts for fulfilling their metabolic requirements [29]. The lack of prophages in the remaining ten various *Treponema* strains could be due to several reasons. Firstly, prophages could have been present but not identified. Identifying a prophage in a bacterial genome can be difficult for many reasons including: (i) a lack of annotation of the bacterial genome (ii) only a few phage-like genes to be found in a short sequence region (iii) only a remnant may be left of a once functional prophage, or (iv) prophages may be undetectable within a bacterial genome that is considered fully annotated but incorrectly so [49]. Another explanation could be that by chance, individuals with no phage genomes could have been chosen for sequencing [9]. A third explanation is that no prophages are present in those bacterial genomes, as a common finding seems to be that only around 50% of bacterial species analysed have been found to be lysogens [50, 51].

When seeking to identify potential hypotheses to account for the absence of prophages in thirteen of the *Treponema* genomes, no apparent patterns were identified, as have been seen in previous studies [50, 51], such as minimum doubling time of the host, genome size, CRISPR-Cas systems or pathogenicity. The *T. pallidum* genomes are small, at 1.1 MB in length and have no CRISPR-Cas systems, however, they are pathogens. The remaining ten *Treponema* genomes without prophages are of a similar size to the lysogens, and all contain CRISPR-Cas systems bar *T. vincentii*, suggesting divergence in these correlations across bacterial taxa. However, several of the strains that appeared devoid of prophages here were single representatives of their species and therefore prophages within the wider species cannot be ruled out.

There were several limitations of this study, including only being able to investigate a subset of *Treponema* genomes and the limitation of using prophage identification software that has been developed or trained on known phages. Prophage integrase genes are always adjacent or very near the attachment site on the phage chromosome, so can typically mark one end of the integrated prophage [9, 52]. However, it can be difficult to distinguish the actual end of the prophage and start of the bacterial genome. Here we double checked the geNomad results manually to estimate the beginning and end of each prophage region as accurately as possible, as phage genomes show distinct gene clustering according to general function [9].

## Conclusions

In this study, we describe 38 prophage-like sequences present in 24 *Treponema* genomes substantially increasing the foundation knowledge of phages infecting Treponemal species. The majority of the 38 prophage regions appear to be distinct from any other described bacteriophages to date and have presented strong evidence for the presence of prophages with high diversity as well as three distinct prophage region clusters within *T. phagedenis* strains, as confirmed by four independent analyses. This data will aid in future characterisation of potential treponemal prophages in existing and future genome and metagenomic datasets. The data also demonstrates compelling evidence for the presence of several potentially functional prophages and that further research could identify prophages which have the potential to be therapeutic agents against a medically important genus for both humans and animals.

## Methods

### Detection of prophages in *Treponema* species

Representative *Treponema* species with complete genome sequences and valid GenBank accession numbers that could be obtained from the RefSeq database (https://www.ncbi.nlm.nih.gov/refseq/. (accessed on 11 December 2022)) were analysed, which led to a total of twenty-four complete *Treponema* genome sequences. These were screened for the presence of prophages using PHASTER (PHAge Search Tool Enhanced Release) [53], PHASTEST (PHAge Search Tool with Enhanced Sequence Translation) [54] and geNomad v1.7.4 [55], using end to end modules and default options. Each bacterial genome was also manually inspected using Artemis v18.2.0 [56], a genome browser that allows visualization of sequence features. Each genome was surveyed for areas that could be identified as potential prophage regions, based on the following criteria: (i) identifying reasonably conserved phage proteins already annotated, such as integrases, portal proteins, terminases, tail tape measure proteins [10], (ii) consecutive hypothetical proteins, (iii) putatively co-transcribed and contiguous open reading frames (iv) encoded within the same DNA strand [52]. The beginning and end of the prophage sequences were estimated by geNomad as well as by manual estimation using (i) the presence of integrases [52], (ii) recognizing when genes started to be annotated again and were likely bacterial in origin and (iii) observing when the genes started to cross the DNA strands again. The identified possible prophage like sequences were subsequently saved and CheckV [57] was used to assess the quality of the viral genomes. Any sequences with no viral genes detected were removed from the study.

### Prophage annotation and morphological classification

All prophage sequences were annotated with Pharokka v1.5.0 [58]. Specifically, coding sequences (CDS) were predicted with PHANOTATE v1.5.0 [59], tRNAs were predicted with tRNAscan-SE v2.0 [60], tmRNAs were predicted with Aragorn v1.2.38 [61] and CRISPRs were predicted with CRISPR Recognition Tool v1.1 [62]. Functional annotation was generated by matching each CDS to the PHROGs [63], VFDB [64] and CARD [65] databases using MMseqs2 [66]. Contigs were matched to their closest hit in the INPHARED database [67] using Mash v2.3 [68]. To supplement the annotation process, any hypothetical genes not detected by Pharokka were subjected to manual analysis using UniProt v2023_05 [69] and Basic Local Alignment Search Tool (BLAST) [70].

The determination of phage morphology relied on the presence of specific structural proteins. The presence of a tail sheath protein indicated prophages with myoviral morphology (contractile-tailed phages) [40, 41]. Conversely, the presence of a tail tape measure protein without a tail sheath protein indicated siphoviral morphology [40, 41].

### Prophage genome and proteome analysis

Phylogenetic tree and intergenomic similarity analysis were constructed using VICTOR (viral comparison and tree building online resource, https://ggdc.dsmz.de/victor.php#) [71], and VIRIDIC v1.1 (Virus Intergenomic Distance Calculator) [72]. Proteome analysis and alignments were created using VirClust v2.0 [73] and ViPTree v3.7 [74].

Genomic synteny of the prophage genomes was visualised with the application of Mauve v2.0 [75] and intergenomic comparison diagrams were created using Clinker v0.0.27 [76] using default setting. PADLOC web server (PADLOC v1.2.0 and padlocdb v1.5.0) [77] were used to identify anti-viral defence mechanisms and UniProt v2023_05 to identify nearest homologues [78]. PhageLeads [69] was used to identify antimicrobial resistance genes and virulence genes.

### Supplementary Information

Additional file 1. Details of Treponema genomes examined and associated identified putative prophage regions. The table shows the results of the prophages identified from the search of 24 different* Treponema* genomes

Additional file 2. 27087P2 additional annotations. The file shows the results from UniProt and BLASTp for the hypothetical protein coding sequences in the prophage sequence

Additional file 3. B43P2 additional annotations. The file shows the results from UniProt and BLASTp for the hypothetical protein coding sequences in the prophage sequence

Additional file 4. KS1P6 additional annotations. The file shows the results from UniProt and BLASTp for the hypothetical protein coding sequences in the prophage sequence

Additional file 5. Annotation summary from Pharokka for the 24 prophage sequences forming three clusters. The table shows the results from Pharokka regarding gene annotation and identification of defence mechanisms.

Additional file 6. B43P2 Padloc. The file shows the Padloc result for the prophage sequence B43P2.

Additional file 7. B43P8 Padloc and UniProt. The file shows the Padloc result for prophage B48P8 and the UniProt results for similarity.

Additional file 8. T320AP1 Padloc and UniProt. The file shows the Padloc result for prophage T320AP1 and the UniProt results for similarity.

Additional file 9. All identified prophage sequences. The file contains all the fasta sequences for the 38 prophage regions identified in this paper.

## Data Availability

The datasets supporting the conclusions of this article are included within the article and its additional files.

## Abbreviations

NCBI: National Centre for Biotechnology Information
DNA: Deoxyribonucleic acid
dsDNA: Double stranded deoxyribonucleic acid
PHASTER: PHAge Seach Tool Enhanced Release
PHASTEST: PHAge Search Tool with Enhanced Sequence Translation
VICTOR: Viral comparison and tree building online resource
VIRIDIC: Virus Intergenomic Distance Calculator
CRISPR-cas: Clustered regularly interspersed palindromic repeats
ICTV: International Committee on Taxonomy of Viruses
PADLOC: Prokaryotic Antiviral Defence LOCator
BLAST: Basic Local Alignment Search Tool

## References

[1] Cooper IR (2016). A review of current methods using bacteriophages in live animals, food and animal products intended for human consumption. J Microbiol Methods.

[2] Hendrix RW, Smith MC, Burns RN, Ford ME, Hatfull GF (1999). Evolutionary relationships among diverse bacteriophages and prophages: All the world’sa phage. Proc Natl Acad Sci USA.

[3] Mushegian A. Are there 1031 virus particles on earth, or more, or fewer?. J Bacteriol. 2020;202(9): 10.1128/jb.00052-20. 10.1128/JB.00052-20 PMC7148134 32071093

[4] Sayers EW, Beck J, Bolton EE, Bourexis D, Brister JR, Canese K (2021). Database resources of the national center for biotechnology information. Nucleic Acids Res.

[5] Zhu Y, Shang J, Peng C, Sun Y (2022). Phage family classification under Caudoviricetes: A review of current tools using the latest ICTV classification framework. Front Microbiol.

[6] Łoś M, Węgrzyn G (2012). Pseudolysogeny. Adv Virus Res.

[7] Hobbs Z, Abedon ST (2016). Diversity of phage infection types and associated terminology: the problem with ‘Lytic or lysogenic’. FEMS Microbiol Lett..

[8] Clokie MR, Millard AD, Letarov AV, Heaphy S (2011). Phages in nature. Bacteriophage.

[9] Casjens S (2003). Prophages and bacterial genomics: what have we learned so far?. Mol Microbiol..

[10] Canchaya C, Proux C, Fournous G, Bruttin A, Brüssow H (2003). Prophage genomics. Microbiol Mol Biol Rev.

[11] Nadeem A, Wahl LM (2017). Prophage as a genetic reservoir: Promoting diversity and driving innovation in the host community. Evolution.

[12] Wagner PL, Waldor MK (2002). Bacteriophage control of bacterial virulence. Infect Immun.

[13] Nagel T, Musila L, Muthoni M, Nikolich M, Nakavuma JL, Clokie MR (2022). Phage banks as potential tools to rapidly and cost-effectively manage antimicrobial resistance in the developing world. Curr Opin Virol.

[14] Kortright KE, Chan BK, Koff JL, Turner PE (2019). Phage therapy: a renewed approach to combat antibiotic-resistant bacteria. Cell Host Microbe.

[15] Lynch KH, Seed KD, Stothard P, Dennis JJ (2010). Inactivation of Burkholderia cepacia complex phage KS9 gp41 identifies the phage repressor and generates lytic virions. J Virol.

[16] Zhang H, Fouts D, DePew J, Stevens R (2013). Genetic modifications to temperate Enterococcus faecalis phage ϕEf11 that abolish the establishment of lysogeny and sensitivity to repressor, and increase host range and productivity of lytic infection. Microbiology.

[17] Brown R, Lengeling A, Wang B (2017). Phage engineering: how advances in molecular biology and synthetic biology are being utilized to enhance the therapeutic potential of bacteriophages. Quant Biol..

[18] Mahler M, Costa AR, van Beljouw SP, Fineran PC, Brouns SJ (2023). Approaches for bacteriophage genome engineering. Trends Biotechnol.

[19] Schuch R, Fischetti VA (2006). Detailed genomic analysis of the Wβ and γ phages infecting Bacillus anthracis: implications for evolution of environmental fitness and antibiotic resistance. J Bacteriol.

[20] Matsuzaki S, Yasuda M, Nishikawa H, Kuroda M, Ujihara T, Shuin T (2003). Experimental protection of mice against lethal Staphylococcus aureus infection by novel bacteriophage ϕMR11. J Infect Dis.

[21] Edgar R, Friedman N, Molshanski-Mor S, Qimron U (2012). Reversing bacterial resistance to antibiotics by phage-mediated delivery of dominant sensitive genes. Appl Environ Microbiol.

[22] Nale JY, Spencer J, Hargreaves KR, Buckley AM, Trzepiński P, Douce GR (2016). Bacteriophage combinations significantly reduce Clostridium difficile growth in vitro and proliferation in vivo. Antimicrob Agents Chemother.

[23] Saheb S (1974). Spirochetal organisms from pigs. 3. Preliminary observations on bacteriophage particles associated with spirochetes of the genus Treponema. Rev Can Biol..

[24] Ritchie A, Robinson I, Joens L, Kinyon J (1978). A bacteriophage for Treponema hyodysenteriae. Vet Rec.

[25] Berthiaume L, Elazhary Y, Alain R, Ackermann H-W (1979). Bacteriophage–like particles associated with a spirochete. Can J Microbiol.

[26] Masuda K, Kawata T (1979). Bacteriophage-like particles induced from the Reiter treponeme by mitomycin C. FEMS Microbiol Lett.

[27] Demirkan I, Williams H, Dhawi A, Carter S, Winstanley C, Bruce K (2006). Characterization of a spirochaete isolated from a case of bovine digital dermatitis. J Appl Microbiol.

[28] Mitchell HL, Dashper SG, Catmull DV, Paolini RA, Cleal SM, Slakeski N (2010). Treponema denticola biofilm-induced expression of a bacteriophage, toxin–antitoxin systems and transposases. Microbiology.

[29] Radolf JD, Deka RK, Anand A, Šmajs D, Norgard MV, Yang XF (2016). Treponema pallidum, the syphilis spirochete: making a living as a stealth pathogen. Nat Rev Microbiol.

[30] Choi B-K, Nattermann H, Grund S, Haider W, Göbel U (1997). Spirochetes from digital dermatitis lesions in cattle are closely related to treponemes associated with human periodontitis. Int J Syst Bacteriol.

[31] Uzochukwu I, Moyes D, Proctor G, Ide M (2023). The key players of dysbiosis in Noma disease; A systematic review of etiological studies. Front Oral Health..

[32] Evans NJ, Brown JM, Demirkan I, Murray RD, Vink WD, Blowey RW (2008). Three unique groups of spirochetes isolated from digital dermatitis lesions in UK cattle. Vet Microbiol.

[33] Demirkan I, Erdoğan M, Demirkan AÇ, Bozkurt F, Altındiş M, Navruz FZ (2018). Isolation and identification of Treponema pedis and Treponema phagedenis-like organisms from bovine digital dermatitis lesions found in dairy cattle in Turkey. J Dairy Sci.

[34] Staton GJ, Clegg SR, Ainsworth S, Armstrong S, Carter SD, Radford AD (2021). Dissecting the molecular diversity and commonality of bovine and human treponemes identifies key survival and adhesion mechanisms. PLoS Pathog.

[35] Ho SFS, Wheeler NE, Millard AD, van Schaik W (2023). Gauge your phage: benchmarking of bacteriophage identification tools in metagenomic sequencing data. Microbiome.

[36] Tu A-HT, Voelker LL, Shen X, Dybvig K (2001). Complete nucleotide sequence of the mycoplasma virus P1 genome. Plasmid..

[37] Lubbers MW, Waterfield NR, Beresford T, Le Page R, Jarvis AW (1995). Sequencing and analysis of the prolate-headed lactococcal bacteriophage c2 genome and identification of the structural genes. Appl Environ Microbiol.

[38] Campoy S, Aranda J, Àlvarez G, Barbé J, Llagostera M (2006). Isolation and sequencing of a temperate transducing phage for Pasteurella multocida. Appl Environ Microbiol.

[39] Turner D, Kropinski AM, Adriaenssens EM (2021). A roadmap for genome-based phage taxonomy. Viruses.

[40] Pell LG, Kanelis V, Donaldson LW, Lynne Howell P, Davidson AR (2009). The phage λ major tail protein structure reveals a common evolution for long-tailed phages and the type VI bacterial secretion system. Proc Natl Acad Sci.

[41] Veesler D, Cambillau C (2011). A common evolutionary origin for tailed-bacteriophage functional modules and bacterial machineries. Microbiol Mol Biol Rev.

[42] Sharma V, Hünnefeld M, Luthe T, Frunzke J (2023). Systematic analysis of prophage elements in actinobacterial genomes reveals a remarkable phylogenetic diversity. Sci Rep.

[43] Buyuktimkin B, Zafar H, Saier MH (2019). Comparative genomics of the transportome of Ten Treponema species. Microb Pathog.

[44] Qian C, Ma J, Liang J, Zhang L, Liang X (2022). Comprehensive deciphering prophages in genus Acetobacter on the ecology, genomic features, toxin-antitoxin system and linkage with CRISPR-Cas system. Front Microbiol.

[45] Fortier L-C, Sekulovic O (2013). Importance of prophages to evolution and virulence of bacterial pathogens. Virulence.

[46] Egido JE, Costa AR, Aparicio-Maldonado C, Haas P-J, Brouns SJ (2022). Mechanisms and clinical importance of bacteriophage resistance. FEMS Microbiol Rev..

[47] Ka D, Oh H, Park E, Kim J-H, Bae E (2020). Structural and functional evidence of bacterial antiphage protection by Thoeris defense system via NAD+ degradation. Nat Commun.

[48] Ofir G, Herbst E, Baroz M, Cohen D, Millman A, Doron S (2021). Antiviral activity of bacterial TIR domains via immune signalling molecules. Nature.

[49] Zhao Y, Wang K, Ackermann H-W, Halden RU, Jiao N, Chen F (2010). Searching for a “hidden” prophage in a marine bacterium. Appl Environ Microbiol.

[50] Ackerman H, DuBow M (1987). Viruses of prokaryotes. Gen Properties Bacteriophages..

[51] Touchon M, Bernheim A, Rocha EP (2016). Genetic and life-history traits associated with the distribution of prophages in bacteria. ISME J.

[52] Buckley D, Odamaki T, Xiao J, Mahony J, van Sinderen D, Bottacini F (2021). Diversity of human-associated bifidobacterial prophage sequences. Microorganisms.

[53] Arndt D, Grant JR, Marcu A, Sajed T, Pon A, Liang Y (2016). PHASTER: a better, faster version of the PHAST phage search tool. Nucleic Acids Res.

[54] Wishart DS, Han S, Saha S, Oler E, Peters H, Grant Jason R (2023). PHASTEST: faster than PHASTER, better than PHAST. Nucleic Acids Res.

[55] Camargo AP, Roux S, Schulz F, Babinski M, Xu Y, Hu B, et al. Identification of mobile genetic elements with geNomad. Nat Biotechnol. 2023:1–0. 10.1038/s41587-023-01953-y. 10.1038/s41587-023-01953-y PMC11324519 37735266

[56] Berriman M, Rutherford K (2003). Viewing and annotating sequence data with Artemis. Brief Bioinform.

[57] Nayfach S, Camargo AP, Schulz F, Eloe-Fadrosh E, Roux S, Kyrpides NC (2021). CheckV assesses the quality and completeness of metagenome-assembled viral genomes. Nat Biotechnol.

[58] Bouras G, Nepal R, Houtak G, Psaltis AJ, Wormald P-J, Vreugde S (2023). Pharokka: a fast scalable bacteriophage annotation tool. Bioinformatics..

[59] McNair K, Zhou C, Dinsdale EA, Souza B, Edwards RA (2019). PHANOTATE: a novel approach to gene identification in phage genomes. Bioinformatics.

[60] Chan PP, Lin BY, Mak AJ, Lowe TM (2021). tRNAscan-SE 2.0: improved detection and functional classification of transfer RNA genes. Nucleic Acids Res..

[61] Laslett D, Canback B (2004). ARAGORN, a program to detect tRNA genes and tmRNA genes in nucleotide sequences. Nucleic Acids Res.

[62] Bland C, Ramsey TL, Sabree F, Lowe M, Brown K, Kyrpides NC (2007). CRISPR recognition tool (CRT): a tool for automatic detection of clustered regularly interspaced palindromic repeats. BMC Bioinformatics.

[63] Terzian P, Olo Ndela E, Galiez C, Lossouarn J, Pérez Bucio RE, Mom R (2021). PHROG: families of prokaryotic virus proteins clustered using remote homology. NAR Genom Bioinform..

[64] Chen L, Yang J, Yu J, Yao Z, Sun L, Shen Y (2005). VFDB: a reference database for bacterial virulence factors. Nucleic Acids Res..

[65] Alcock BP, Raphenya AR, Lau TT, Tsang KK, Bouchard M, Edalatmand A (2020). CARD 2020: antibiotic resistome surveillance with the comprehensive antibiotic resistance database. Nucleic Acids Res.

[66] Steinegger M, Söding J (2017). MMseqs2 enables sensitive protein sequence searching for the analysis of massive data sets. Nat Biotechnol.

[67] Cook R, Brown N, Redgwell T, Rihtman B, Barnes M, Clokie M (2021). INfrastructure for a PHAge REference database: identification of large-scale biases in the current collection of cultured phage genomes. Phage.

[68] Ondov BD, Treangen TJ, Melsted P, Mallonee AB, Bergman NH, Koren S (2016). Mash: fast genome and metagenome distance estimation using MinHash. Genome Biol.

[69] The UniProt Consortium (2017). UniProt: the universal protein knowledgebase. Nucleic Acids Res.

[70] Altschul SF, Gish W, Miller W, Myers EW, Lipman DJ (1990). Basic local alignment search tool. J Mol Biol.

[71] Meier-Kolthoff JP, Göker M (2017). VICTOR: genome-based phylogeny and classification of prokaryotic viruses. Bioinformatics.

[72] Moraru C, Varsani A, Kropinski AM (2020). VIRIDIC—A novel tool to calculate the intergenomic similarities of prokaryote-infecting viruses. Viruses.

[73] Moraru C (2023). VirClust—A tool for hierarchical clustering, core protein detection and annotation of (prokaryotic) viruses. Viruses.

[74] Nishimura Y, Yoshida T, Kuronishi M, Uehara H, Ogata H, Goto S (2017). ViPTree: the viral proteomic tree server. Bioinformatics.

[75] Darling AE, Mau B, Perna NT (2010). Progressive Mauve: multiple genome alignment with gene gain, loss and rearrangement. PLoS ONE.

[76] Gilchrist CL, Chooi Y-H (2021). Clinker & clustermap. js: Automatic generation of gene cluster comparison figures. Bioinformatics..

[77] Payne LJ, Meaden S, Mestre MR, Palmer C, Toro N, Fineran PC (2022). PADLOC: a web server for the identification of antiviral defence systems in microbial genomes. Nucleic Acids Res.

[78] Yukgehnaish K, Rajandas H, Parimannan S, Manickam R, Marimuthu K, Petersen B (2022). PhageLeads: rapid assessment of phage therapeutic suitability using an ensemble machine learning approach. Viruses.

