# Supplementary material for: Genomic and taxonomic evaluation of 38 Treponema prophage sequences
Source: BMC Genomics. 2024 Jun 1;25:549. doi: 10.1186/s12864-024-10461-5 (PMC11144348; doi:10.1186/s12864-024-10461-5)
Supplement: Supplementary file 1 — Additional file 1. Details of Treponema genomes examined and associated identified putative prophage regions. The table shows the results of the prophages identified from the search of 24 different Treponema genomes [file 12864_2024_10461_MOESM1_ESM.docx]

**Details of Treponema genomes examined and associated identified putative prophage regions -** *The table shows the results of the prophages identified from the search of 24 different Treponema genomes.*

| ***Treponema* Group** | ***Treponema* Strain** | **Genbank accession number** | **Origin, genome size + GC %** | **Number of prophage regions** | **Prophage name** | **Location in Bacterial Genome** | **CheckV quality and % completeness** | **Length (kb)** | **CDS No.** | **PHASTEST *att* sites identified** | **GC %** | **% of viral DNA in genome** |
| --- | --- | --- | --- | --- | --- | --- | --- | --- | --- | --- | --- | --- |
| Group 1 | *T. medium* T19 DSM 18689 | GCA_017161225.1 | Bovine digital dermatitis lesion  2.8 Mb  44.3% | 0 |  |  |  |  |  |  |  |  |
|  | *T. medium* ATCC 700293 | GCA_000413035.1 | Human oral  2.7 Mb  44.3% | 0 |  |  |  |  |  |  |  |  |
|  | *T. vincentii* F0403_V1 | GCA_000412995.1 | Human oral  2.3 Mb  45.5% | 2 | VinP1 | 13300-50445 | High-100% | 37.1 | 59 | *att*L = 30569 (13bp)  *att*R = 34326 (13bp)  CCTCATCCTCCTT | 45.6 | 3.1% |
|  |  |  |  |  | VinP2 | 2120911-2157024 | High-100% | 36.1 | 55 | attL = 2137657 (13bp)  attR = 2155425 (13bp)  CAGCCTGACTATA | 44.9 |  |
| Group 2 | *T. phagedenis* T320A DSM 18690 | GCA_017161245.1 | Bovine digital dermatitis lesion  3.1 Mb  39.9% | 3 | T320AP1 | 143495-208808 | Medium-80% | 65.3 | 88 |  | 39.6 | 7.4% |
|  |  |  |  |  | T320AP2 | 1299566-1353585 | Low-41% | 54 | 71 |  | 38.9 |  |
|  |  |  |  |  | T320AP3 | 1843908-1897027 | Low-48% | 53.1 | 97 |  | 38.8 |  |
|  | *T. phagedenis* B43.1 | GCA_008153345.1 | Bovine digital dermatitis lesion  3.4 Mb  39.5% | 8 | B43P1 | 22621-63230 | Low-42% | 40.6 | 61 |  | 40.1 | 12.8% |
|  |  |  |  |  | B43P2 | 470999-517852 | Low-44% | 46.8 | 74 |  | 39.8 |  |
|  |  |  |  |  | B43P3 | 826510-862631 | Low-43% | 36.1 | 43 |  | 39.2 |  |
|  |  |  |  |  | B43P4 | 1159207-1232776 | High-91% | 73.5 | 81 |  | 39.8 |  |
|  |  |  |  |  | B43P5 | 1837003-1910060 | High-91% | 73 | 81 |  | 39.4 |  |
|  |  |  |  |  | B43P6 | 2021514-2069190 | Medium-61% | 47.6 | 77 | *att*L = 3049472 (14bp)  attR = 3073625 (14bp)  TTTTGAAAAGCTTA | 39.2 |  |
|  |  |  |  |  | B43P7 | 2640576-2704816 | Medium -73% | 64.2 | 84 |  | 40.9 |  |
|  |  |  |  |  | B43P8 | 3049191-3102895 | Medium- 64% | 53.6 | 87 |  | 37.7 |  |
|  | *T. phagedenis* KS1 | GCA_013348685.1 | Bovine digital dermatitis lesion  3.2 Mb  40% | 8 | KS1P1 | 22621-56058 | Low-40% | 33.4 | 57 |  | 39.9 | 12.4% |
|  |  |  |  |  | KS1P2 | 458135-511005 | Low-36% | 52.9 | 93 |  | 39.7 |  |
|  |  |  |  |  | KS1P3 | 795318-829302 | Low-40-% | 33.9 | 56 |  | 39.5 |  |
|  |  |  |  |  | KS1P4 | 1104571-1169486 | Medium-60% | 64.9 | 102 |  | 39.3 |  |
|  |  |  |  |  | KS1P5 | 1739431-1805363 | Medium-81% | 65.9 | 93 |  | 39.5 |  |
|  |  |  |  |  | KS1P6 | 1916504-1960784 | Medium-57% | 44.2 | 72 |  | 39.8 |  |
|  |  |  |  |  | KS1P7 | 2484324-2546506 | Medium-74% | 62.1 | 95 | *att*L = 2896535 (14bp)  *att*R = 2920519 (14bp)  TTTTGAAAAGCTTA | 40.8 |  |
|  |  |  |  |  | KS1P8 | 2896254-2935835 | Low-45% | 39.5 | 75 |  | 39.2 |  |
|  | *T. phagedenis* Reiter | GCA_017161285.1 | Human  2.9 Mb  40.2% | 2 | ReiterP1 | 497075-549603 | Medium-63% | 52.5 | 75 |  | 40 | 4.4% |
|  |  |  |  |  | ReiterP2 | 706841-781896 | Medium-87% | 75 | 171 |  | 39.5 |  |
|  | *T. phagedenis* 27087 | GCA_008271205.1 | Human  3.5 Mb  40% | 4 | 27087P1 | 70425-105815 | Low-42% | 35.3 | 51 |  | 39.6 | 6.5% |
|  |  |  |  |  | 27087P2 | 190311-257900 | Medium-84% | 67.6 | 88 |  | 40 |  |
|  |  |  |  |  | 27087P3 | 505373-578592 | High-91% | 73.2 | 98 |  | 39.5 |  |
|  |  |  |  |  | 27087P4 | 2737677-2790598 | Medium-67% | 52.9 | 86 |  | 38.8 |  |
| Group 3 | *T. pedis* T3552B DSM 18691 | GCA_017161325.1 | Bovine digital dermatitis lesion  2.9 Mb  36.9% | 0 |  |  |  |  |  |  |  |  |
|  | *T. pedis* KS1 | GCA_015219785.1 | Bovine digital dermatitis lesion  3.0 Mb  36.8% | 0 |  |  |  |  |  |  |  |  |
|  | *T. pedis* A4 | GCA_000447675.1 | Porcine  2.9 Mb  36.9% | 0 |  |  |  |  |  |  |  |  |
|  | *T. denticola ATCC 35405* | GCA_000008185.1 | Human oral  2.8 Mb  37.9% | 1 | td1 | 1164843-1201347 | Medium-68% | 36.5 | 41 | *att*L = 1175219 (16bp)  *att*R = 1214694 (16bp)  AACTCCCGCATAGCCT | 36.9 | 1.3% |
|  | *T. putidum* OMZ 758 | GCA_000755145.1 | Human oral  2.8 Mb  37% | 0 |  |  |  |  |  |  |  |  |
| Bovine Gastrointestinal tract (GIT) | *T. rectale* CHPA | GCA_014984185.1 | Bovine rectum  2.8 Mb  39% | 0 |  |  |  |  |  |  |  |  |
|  | *T. ruminus* RU1 | GCA_017161305.1 | Bovine rumen  2.8 Mb  44.6% | 1 | RuP1 | 262571-325613 | High-100% | 63 | 63 |  | 44.5 | 2.2% |
|  | *T. bryantii* B25 | GCA_900111035.1 | Bovine rumen  3.4 Mb  38% | 1 | BryP1 | 490640-562747 | Medium-89% | 72.1 | 149 |  | 37.2 | 2.1% |
|  | *T. socranskii* ATCC 35535 | GCA_024181585.1 | Human oral  2.0 Mb  48.5% | 0 |  |  |  |  |  |  |  |  |
| Insect Gastrointestinal tract (GIT) | *T. azotonutricium* ZAS-9 | GCF_000214355.1 | Termite GIT  3.8 Mb  49.5 | 4 | AzoP1 | 412972-457572 | Medium-55% | 44.6 | 74 | *att*L = 438242 (12bp)  *att*R = 462327 (12bp)  GTATTGATAAAG | 47 | 3.8% |
|  |  |  |  |  | AzoP2 | 1372512-1429291 | Medium-73% | 56.7 | 69 | *att*L = 1389833 (15bp)  *att*R = 1417047 (15bp)  ACCTTTGTGAAATAT | 45 |  |
|  |  |  |  |  | AzoP3 | 1869218-1902392 | Low-22% | 33.1 | 50 |  | 49 |  |
|  |  |  |  |  | AzoP4 | 3708494-3720963 | Low-18% | 12.4 | 27 |  | 47.8 |  |
|  | *T. primitia* ZAS-2 | GCA_000214375.1 | Termite GIT  4.1 Mb  50.5% | 4 | PrimP1 | 2101786-2132061 | Low-26% | 17.9 | 32 |  | 44 | 3.2% |
|  |  |  |  |  | PrimP2 | 2099859-2132121 | Low-22% | 30.2 | 43 |  | 56.4 |  |
|  |  |  |  |  | PrimP3 | 2181654-2225148 | Medium-56% | 43.4 | 71 |  | 47.6 |  |
|  |  |  |  |  | PrimP4 | 3415045-3455582 | Low-35% | 40.5 | 54 |  | 49.6 |  |
| Miscellaneous | *T. caldarium* DSM 7334 | GCF_000219725.1 | Environmental  3.2 Mb  45.5% | 0 |  |  |  |  |  |  |  |  |
|  | *T. maltophilum* ATCC 51939 | GCA_000413055.1 | Human oral  2.5 Mb  47.5% | 0 |  |  |  |  |  |  |  |  |
|  | *T. pallidum subsp pallidum Nichols* | GCA_000008605.1 | Human syphilis  1.1 Mb  52.8% | 0 |  |  |  |  |  |  |  |  |
|  | *T. pallidum pertenue* LMNP-1 | GCA_003076575.1 | Human Yaws   - 1. Mb   52.5% | 0 |  |  |  |  |  |  |  |  |
|  | *T. paraluiscuniculi cuniculi A* | GCA_000217655.1 | Rabbit syphilis  1133390 bp   - 1. Mb   52.7% | 0 |  |  |  |  |  |  |  |  |
